# A Composite Material Repair Structure: For Defect Repair of Branch Pipe Fillet Welds in Oil and Gas Pipelines

**DOI:** 10.3390/ma19020222

**Published:** 2026-01-06

**Authors:** Liangshuo Zhao, Yingjie Qiao, Zhongtian Yin, Bo Xie, Bangyu Wang, Jingxue Zhou, Siyu Chen, Zheng Wang, Xiaodong Wang, Xiaohong Zhang, Xiaotian Bian, Xin Zhang, Yan Wu, Peng Wang

**Affiliations:** 1 College of Materials Science and Chemical Engineering, Harbin Engineering University, Harbin 150001, Chinashepherd@hrbeu.edu.cn (B.X.); zhoujingxue@hrbeu.edu.cn (J.Z.); wangxiaodong@hrbeu.edu.cn (X.W.); zhangxiaohong@hrbeu.edu.cn (X.Z.); bxt0615@hrbeu.edu.cn (X.B.); 15945227533@163.com (X.Z.);; 2 School of Materials Science and Engineering, China University of Petroleum (East China), Qingdao 266580, China; wangbangyu222@163.com (B.W.); 20230026@upc.edu.cn (P.W.)

**Keywords:** composite repair, branch pipe fillet weld, finite element analysis, bursting test, pipeline maintenance

## Abstract

In the oil and gas pipeline industry, numerous small-diameter branch pipe fillet welds exist, which are prone to stress concentration because of diverse geometric shapes. The internal welding defects within these welds pose severe hazards to safe production. Specifically, the irregular geometry often leads to internal root defects where the weld metal fails to fully penetrate the joint or fuse with the base material (referred to as incomplete penetration and incomplete fusion). This study developed a GF-CF-GF (CF is carbon fiber, GF is glass fiber) sandwich composite reinforcement structure for pipe fittings with these specific internal defects (main pipe: Φ323.9 × 12.5 mm; branch pipe: Φ76 × 5 mm) through a combination of finite element analysis (FEA) and burst test verification. The inherent correlation between structural factors and pressure-bearing capacity was revealed by analyzing the influence of defect sizes. Based on FEA, the repair layer coverage should be designed to be within 400 mm from the defect along the main pipe wall direction and within 100 mm from the defect along the branch pipe wall direction, with required thicknesses of 5.6 mm for incomplete penetration and 3.2 mm for incomplete fusion. Analysis of the actual burst test pressure curve showed that the elastic-plastic transition interval of the repaired pipes increased by approximately 2 MPa compared to normal undamaged pipes, and their pressure-bearing capacities rose by 1.57 MPa (incomplete penetration) and 1.76 MPa (incomplete fusion). These results demonstrate the feasibility of the proposed reinforcement design, which has potential applications in the safety and integrity of oil and gas transportation.

## 1. Introduction

In many fields, including oil and gas pipelines, petrochemical, and power industries, there exist numerous small-diameter branch pipe fillet welds [1,2], and these small-diameter branch pipe fillet welds are usually subjected to multiple complex conditions such as high pressure, high temperature, and corrosive media. In addition, due to their irregular geometric shapes, during the long-term service of oil and gas transmission pipelines, the diverse stress forms of the fillet welds easily cause local stress concentration and tend to generate welding defects such as incomplete penetration and strip-shaped slag inclusion, which become the starting points of corrosion and cracking [3]. Once leading to pipeline failure, it will seriously affect safe production [4]. Currently, in terms of defect repair technologies, the existing ones are highly homogenized. From the perspective of classification, these technologies are mainly divided into welding-based and non-welding-based according to repair processes: welding-based repair includes overlay welding, patch welding, hot tapping under pressure, pipe replacement, sleeve, and mechanical fixture; non-welding-based repair includes steel epoxy sleeve, grinding, and composite material reinforcement [5,6,7]. After excessive defects or leakage are found in small-diameter branch pipe fillet welds, they are generally repaired by shutting down pipeline operation, cutting out the weld for re-welding, or pipe replacement. However, such repair methods involve high costs and have a significant impact on the normal production of the station, and many small-sized pipelines are not suitable for welding and sleeving. In composite material reinforcement, the stress acting on the damaged part of the pipeline is uniformly transferred to the composite repair layer through the high-strength filler applied to the defect area and the high-strength adhesive between the pipe body and fiber material layers because of the high strength and high modulus of the composite repair layer. Furthermore, the non-hot work operation in oil and gas pipeline operations is of great significance for safe production.

In the field of using the composite material reinforcement method to repair fillet welds of oil and gas pipeline branch pipes, Zhou et al. [8] have already conducted a repair process design for surface defects of fillet welds. However, in composite reinforcement, the internal defects of fillet welds—incomplete fusion and root incomplete penetration—are more critical [8]. Unlike surface defects, internal defects in small-diameter branch pipes pose greater challenges due to the complex stress concentration at the saddle-shaped intersection and the difficulty in determining the effective load transfer mechanism of the repair layer. Furthermore, there is currently a lack of systematic theoretical analysis and experimental verification regarding the optimal structural design and precise parameter determination for repairing these specific internal defects. For internal defects of fillet welds, the welding-based repair method requires shutting down pipeline operation for re-welding, which incurs high repair costs and has a significant impact on normal production. The composite material reinforcement repair method is expected to achieve effective structural restoration of internal defects in small-diameter branch pipe fillet welds, and compared with traditional steel material repair methods such as sleeving repair and patch welding repair, it possesses excellent durability, fatigue resistance, and corrosion resistance [9]. Using composite materials for in situ repair not only reduces costs and increases efficiency but also effectively avoids the massive carbon emissions and environmental burdens associated with manufacturing new steel pipes [10]. In summary, conducting research on the in-service composite material reinforcement repair method for internal defects of small-diameter branch pipe fillet welds and establishing a set of detection and evaluation methods for repair quality are of great significance for ensuring the safe service of small-diameter branch pipes and the stable operation of oil and gas stations.

The theoretical research on the in-service composite material reinforcement repair method for defects of small-diameter branch pipes usually adopts numerical simulation methods. The Abaqus finite element simulation software [11] is used to establish a simulation analysis model for composite-repaired branch pipe fillet welds. Carbon fiber (CF) fabrics and glass fiber (GF) fabrics are selected as reinforcements, with epoxy resin as the matrix. The hand lay-up wet winding process [12,13] is adopted to repair the fillet welds with internal defects of branch pipes. In terms of progressive damage evolution, researchers, on the basis of accounting for the nonlinear behavior of thermoplastic materials, combine the three-dimensional elasticity theory with the Hashin progressive damage criterion [14,15]. A progressive damage failure model combining composites with metal pipes has been established. For the composite damage evolution model, multiple failure criteria such as ultimate pressure-bearing capacity, the Von Mises yield criterion [16] (a finite element yield criterion), and the fourth strength theory [17] are usually adopted as judgment criteria. It controls the stiffness degradation coefficient through main failure modes, which has been widely used in the failure evaluation of composites, and its reliability has been verified through experiments. To address the influence of actual working conditions and the pressure on composite repair, the inherent correlation among material factors, structural factors, and process factors should be established by comparing the repair effects of different repair process parameters, and the measures of improving repair effects, feasibility analysis and strategy research on branch pipe fillet weld repair technology should be proposed. Full-scale specimens with typical defects were fabricated. After completing the repair using the optimized repair technology, burst tests [18] were carried out to evaluate the repair effects of the composites. During the burst tests, strain gauges were used to monitor the stress variation trend near the defects, so as to verify and modify the numerical simulation results.

Therefore, this paper adopted the method of experiments and numerical simulation to provide a repair design method, parameter calculation method, and repair effect evaluation method for composite repair of internal defects in branch pipe fillet welds. It systematically investigated the failure behavior and failure mechanism of fiber-reinforced pipes under burst load, and obtained the stress distribution and its variation law of the repair interface.

## 2. Experimental and Simulation

### 2.1. Simulation

**Figure 1:**
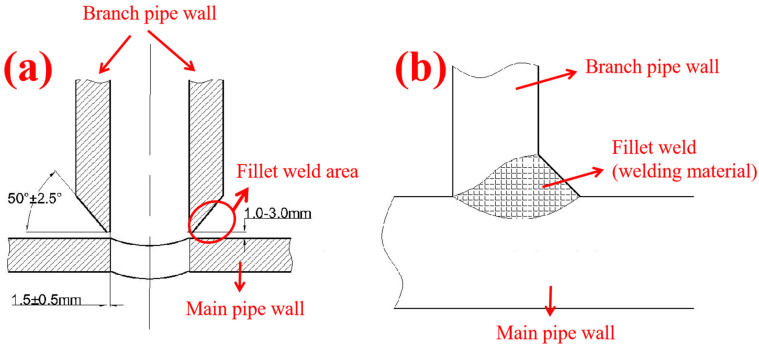
Schematic of Placement Welding Method Configuration. (**a**) Schematic Diagram of an Installed Branch Pipe Structure, (**b**) Schematic Diagram of Fillet Weld Structure.

In this work, we adopted the Abaqus finite element simulation software [19] (version 2020). For the specifications of the repaired fillet weld pipes in the simulation, typical specifications of oil and gas transmission pipelines were selected: the main pipe specification was Φ323.9 × 12.5 mm, and the branch pipe specification was Φ76 × 5 mm. In the established model, the welding process adopted the fillet welding method as shown in Figure 1 [20], with a single-sided V-groove at the welding joint. To account for extreme working conditions, the groove thickness was set to 3 mm. To avoid the structural influence of the pipeline, the model length was designed to be ten times the pipe diameter. The connection between the pipeline and the weld was set as a tie constraint to simulate the welding effect, where the pipeline contact surface was defined as the Main surface and the weld contact surface as the Secondary surface. During the simulation setup, only the internal pressure load (operating load of 12 MPa) was considered in the numerical simulation process. Although the experimental hydrostatic test included pressure-holding stages to verify sealing tightness, the simulation focused on the quasi-static ultimate bearing capacity. Therefore, time-dependent behaviors such as creep and stress relaxation were neglected, and the loading was simplified to a monotonic pressure increase until failure. The base metal of the main pipe was L360, and that of the branch pipe was L245. In accordance with the strong matching principle, the welding material adopted L360, the same as that of the main pipe [21]. The material properties were shown in Table 1. The elastic modulus of CF is close to that of steel, which is conducive to the cooperative deformation between the repair layer and steel pipes, enabling uniform stress distribution and excellent repair effect. However, it is also necessary to consider preventing GF from conducting electricity, which would cause galvanic corrosion between GF and steel [22]. Therefore, the composite material adopted a GF-CF-GF sandwich structure design composed of T700-CF fabrics and S-GF cloth. To ensure the effective transmission of torsional stress and shear stress, as well as the circumferential constraint strength, the winding was set to be wrapped sequentially at angles of 45°/0°/−45°/90°. Considering the working condition environment, the curing temperature was set to 35 °C. We fabricated several composite tensile specimens with different process parameters and performed tensile tests on the specimens using a universal testing machine. The tensile test was conducted according to ASTM D3039 standard [23] using a LE-3000 universal testing machine manufactured by Shanghai Lishi Scientific Instrument Co., Ltd. (Shanghai, China) at a speed of 2 mm/min. The specimen size was 250 × 25 × 3 mm, tested at 25 °C, with a gauge length of 150 mm. The tensile tests revealed that the GF-CF-GF composite exhibits a linear–elastic stress–strain relationship typical of thermoset composites, with a brittle failure mode dominated by fiber breakage and matrix cracking. The measured longitudinal tensile strength of 487 MPa (Table 2) indicates a load-bearing capacity significantly higher than the yield strength of the pipeline steel (L245/L360). This material property serves as the fundamental basis for the repair mechanism: the high-strength composite layer remains in the elastic stage and provides effective confinement even when the steel pipe enters plastic deformation during the burst test. After obtaining the composite properties, finite element repair analysis was conducted to determine the influence of the repair layer’s structural parameters on the repair effect. The composite properties are shown in Table 2.

The carbon fiber used in this study was SYT49S-12K type produced by Shandong Dezhou United Top Composite Materials Co., Ltd. (Dezhou, China). The glass fiber was S-type glass fiber fabric produced by Nanjing Glass Fiber Research and Design Institute Co., Ltd. (Nanjing, China). The epoxy resin and epoxy resin curing agent were both produced by Shanghai Weibo New Materials Technology Co., Ltd. (Shanghai, China), with models T-5089A and 5089B-4 respectively. The structural adhesive was HY-2012-21B type structural adhesive produced by Qingdao Sitangbach Technology Co., Ltd. (Qingdao, China). The acetone cleaner was AD-1 type acetone cleaner produced by Guangdong Taishan Youshun Chemical Co., Ltd. (Taishan, China). ABAQUS CAE 2020 was used as the software employed.

**Figure 2:**
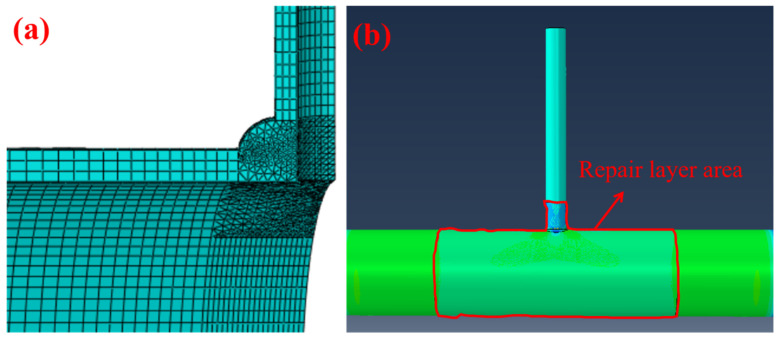
(**a**) Grid division diagram; (**b**) Distribution map of composite layer repair area.

The structure of the fillet weld and its surrounding area was complex, so tetrahedral meshes with mesh refinement were adopted. The mesh size was adjusted according to the actual situation to approximately 1–2 mm, while other parts were divided into hexahedral elements, gradually increasing from the weld side to the end far from the weld, as shown in Figure 2a. To calculate the ultimate pressure-bearing capacity, the analysis step adopted the dynamic explicit algorithm. The composite repair layer was established with shell elements using the Composite Layup module for simulation, with 3 integration points set. The shell reference surface and offset were the bottom surface, and the composite layer was connected to the pipe body through tie constraints. In accordance with the industry standard GB/T 36701-2018 [24], the repair layer area was defined as follows: the boundary of the repair layer was within 100 mm from the defect in the direction of the branch pipe wall, and within 400 mm from the defect in the direction of the main pipe wall. The schematic diagram of the Composite Layup area is shown in Figure 2b. The weld and the pipe were different components constrained by tie constraints, where the pipe contact surface was the Main surface and the weld contact surface was the Secondary surface. The boundary condition at the pipe end was set to fully fixed (U1 = U2 = U3 = UR1 = UR2 = UR3 = 0). Other setting parameters are shown in Table 3.

**Figure 3:**
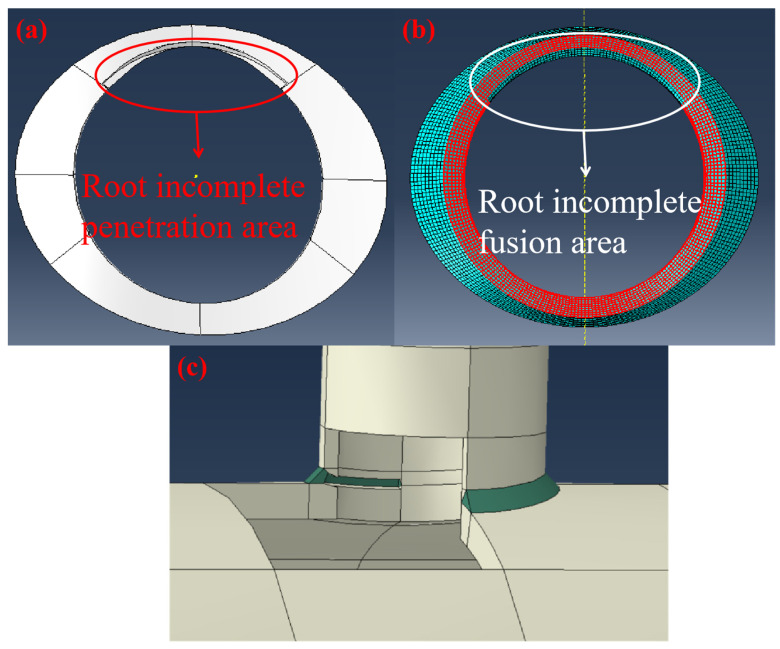
(**a**) Schematic diagram of fillet weld root incomplete penetration setup model and area; (**b**) Schematic diagram of branch pipe root incomplete fusion setup area; (**c**) Macro schematic diagram of design model for incomplete penetration defect of fillet weld.

Incomplete fusion defects are area-type defects formed during the welding process when the weld metal is not fully melted and bonded with the base metal or adjacent weld beads. Thus, in the simulation, partial areas between the branch pipe and the weld were set as not tied. Incomplete penetration defects refer to the phenomenon where the base metal is not melted during welding and the weld metal does not penetrate into the joint root. Incomplete penetration defects occur when the welded metal is not fully fused. Therefore, they still possess some minor strength. However, for industrial safety margin considerations, the strength of the incomplete penetration area needs to be set to 0 in order to better account for the strength loss caused by various types of incomplete penetration defects. Therefore, in the simulation, the root of the weld model needed to be hollowed out. Both defects were set at the inner intersection of the main pipe and the branch pipe to simulate this extreme working condition, as this was the stress concentration area during pipeline operation. The specific defect settings are shown in Figure 3.

The purpose of establishing the analysis model was: calculating the influence of different structural parameters of the repair layer on the repair effect; taking the depth and length of the incomplete fusion and incomplete penetration areas as separate variables to analyze the influence of these two dimensions on actual working conditions and determine the main influencing factors; determining the repairable size and the required minimum thickness of the composite repair layer for root incomplete penetration and root incomplete fusion of the branch pipe.

In the calculation, the criteria were set as follows: the internal angle, stress distribution near the weld, location and value of the maximum stress, and ultimate burst pressure under 1.5 times the operating pressure load. A comparative analysis was conducted on the equivalent plastic strain (PEEQ), location of the maximum stress, Mises stress and its distribution, and the magnitude of the ultimate burst pressure between the two cases. This was to determine whether the stress in the defect area was reduced to a safe range and whether the deformation of the branch pipe was effectively controlled, thereby confirming the repair effect.

### 2.2. Experimental

#### 2.2.1. Sample Preparation

To avoid structural influence, the main pipe of the specimen was 2.2 m long and the branch pipe was 0.4 m long. Pipe heads were welded at the pipe boundaries for sealing, and the pipe specimens were manufactured by Shenyang Zhongsheng Mechanical Equipment Co., Ltd. (Shenyang, China). During the full-size pipeline specimen preparation process, incomplete penetration and incomplete fusion prefabricated welding defects were intentionally adopted [23]. These defects were fabricated to target the nominal dimensions consistent with the FEA model: a depth of 2.0 mm and a circumferential length of 52 mm, representing typical severe defects found in field inspections.

#### 2.2.2. Repair Process

**Figure 4:**
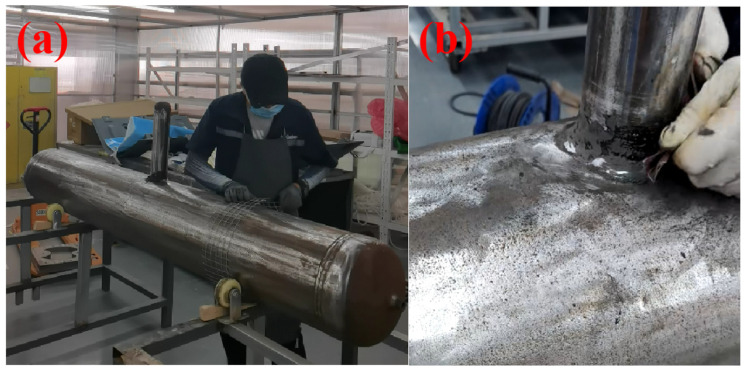
(**a**) Schematic diagram of winding fixture setting; (**b**) Schematic diagram of surface filling treatment for weld seam.

**Figure 5:**
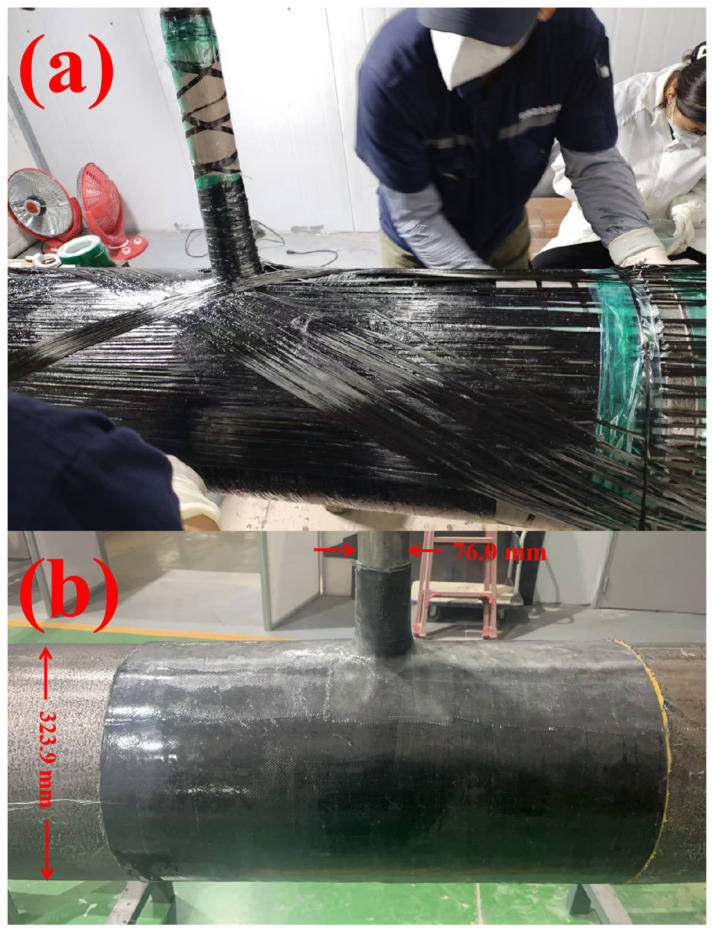
(**a**) CF cloth wrapping repair process(**b**) Schematic of post-repair effect by CF wrapping.

The repair process for defects in pipe fillet welds included the following points:

(1) Winding tooling was set up: to ensure the fiber tape was wound under a certain tension, an iron mesh was installed outside the main pipe repair area. The iron wires at the edges of the iron mesh were bent into a barb shape, so that the fiber tape could hook onto the iron wires at the edges of both ends during winding and be wound back and forth under a certain tension. The details are shown in Figure 4a.

(2) Pretreatment of fillet weld repair: Before repair, strict derusting treatment was performed on the surface. The derusting grade shall meet Sa2.5 or St3 specified in GB/T8923.1-2011 [23], and the surface should be polished smooth and even (Ra ≤ 15 μm) to ensure good adhesion between the repair material and the substrate, thus creating microstructures conducive to mechanical interlocking. During the entire pretreatment process, clean gauze dipped in acetone cleaning agent was used to completely remove oil stains, moisture and other impurities, so as to improve the adhesion and durability of the repair layer. This rigorous surface preparation ensures effective wetting of the resin on the steel substrate, facilitating chemical bonding through the polar groups of the epoxy matrix. The combination of mechanical interlocking and chemical bonding constitutes the primary binding mechanism at the interface. For uneven areas on the fillet weld surface, as well as parts of the pipe with dents, corrosion pits or other defects, HY-2012-21 structural adhesive (filling putty material) produced by Qingdao Sitanhaba Technology Co., Ltd. (Qingdao, China). was adopted. Agent A and Agent B of the adhesive were mixed at a volume ratio of 2:1, and iron powder accounting for 1% of the structural adhesive’s volume was added. The mixture was stirred mechanically/manually for 3–5 min until a uniform color was achieved without streaks. Then, the mixture could be used for filling and leveling treatment, as shown in Figure 4b. The filled area should be polished after curing at room temperature for 1 h to ensure that the height difference is less than 0.5 mm and there are no sharp edges.

(3) Laying glass fiber cloth: During the wrapping process, first apply primer resin to ensure that the epoxy resin fully covers the repair area. The epoxy resin and curing agent were mixed at a ratio of 3:10, 10–30 min before the winding work. Then, starting from the weld, the glass fiber tape with a thickness of at least 0.4 mm was laid as an insulating layer, ensuring full and intimate contact between the glass fiber cloth and the pipe surface. The insulating layer laid in the winding repair area shall ensure full area coverage with no exposure of the base material. After laying the glass fiber tape, the coating operation of the first-layer mixture of epoxy resin and curing agent was immediately performed to ensure that the resin and curing agent fully penetrated the fiber layer, forming a dense and uniform composite structure. During the coating process, tools such as scrapers and rollers shall be used to make the resin uniformly distributed, and entrapped air bubbles shall be removed in a timely manner to prevent defects such as hollowing and delamination from affecting the repair quality. To accelerate the initial cross-linking of the resin, the first layer was assistively cured by using a hot air gun set at 35 °C for at least 2 h. This process promoted gelation and surface drying, resulting in tighter adhesion between the repair layer and the original pipe. The resin and curing agent adopted are T-5089A epoxy resin and LT-5089B-4 curing agent, respectively, produced by Huibo New Material Technology (Shanghai) Co., Ltd. (Shanghai, China).

(4) After the initial curing of the first layer of resin, the epoxy resin and curing agent were mixed at a ratio of 3:10 10–30 min before the carbon fiber winding work. When winding the carbon fiber, starting from the weld, the carbon fiber cloth was wound at multiple angles in the sequence of 90°, 45° and −45°. The resin coating for each layer must uniformly penetrate the fibers, and scrapers or rollers were used to remove air bubbles to avoid delamination. In the complex area where the main pipe and branch pipe connect, a cross-winding method was adopted, mainly at ±45° combined with 90° winding. For the triangular area formed by the branch pipe and main pipe that was difficult to cover due to the small-diameter branch pipe, upright posts were installed to wind the pipe in the 0° ± 5° direction, and additional carbon fiber cloth was laid for reinforcement. The winding tension was controlled at 12 N ± 0.5 N using a pointer-type tension meter. The dynamometer uses the SEG-20-1 model produced by Shanghai Shangcen Precision Instruments Co., Ltd. (Shanghai, China). (which must be calibrated before use) to ensure that the fibers closely adhere to the glass fiber insulating layer on the pipe surface. After laying each 0.8–1.2 mm-thick carbon fiber layer, the coating operation of the epoxy resin and curing agent mixture must be performed immediately to ensure the resin and curing agent fully penetrate the fiber layer, forming a dense and uniform composite structure. During the coating process, tools such as scrapers and rollers shall be used to distribute the resin evenly, and entrapped air bubbles shall be removed in a timely manner to prevent defects like hollowing and delamination from affecting the repair quality. Then, a hot air gun set at 35 °C was used for heating-assisted curing of the layer for no less than 2 h to accelerate the initial cross-linking reaction of the resin and achieve gel surface drying, the hot air gun uses the DH-HG2 model produced by Delixi Group Co., Ltd. (Hangzhou, China). Sealing tape was wrapped around the surface to prevent resin dripping during curing. After complete curing for no less than 24 h, the sealing tape was removed before laying the next carbon fiber layer. Finally, an ultrasonic detector was used to inspect the thickness of the carbon fiber repair layer: one test point every 50 mm in the core repair area and one every 100 mm in the non-core area, the ultrasonic detector uses the CTS-9000 series produced by Shantou Ultrasound Electronics Co., Ltd. (Shantou, China). If the thickness was insufficient: if there was no hollowing or delamination in the existing fiber layer of the corresponding area, direct supplementary winding was performed; if hollowing existed (showing indentation and rebound when pressed with a finger), a small opening was cut along the edge of the hollowed area with a blade, a small amount of resin and curing agent mixture was injected, and a pressure roller was used to press out air bubbles, The pressure roller uses the CF-R series carbon fiber press roller produced by Nantong Zhongding Composite Materials Co., Ltd. (Nantong, China). After standing for 30 min until the resin was semi-cured(loss of fluidity, becomes gel-like, surface sticky), supplementary winding was carried out. The thickness of the carbon fiber layer should be not less than the designed thickness. Figure 5 shows the carbon fiber winding during the repair process and the surface effect after the repair.

Laying surface glass fiber tape: After winding the completion of the carbon fiber layer, glass fiber tape with a thickness of not less than 0.4 mm was laid on the surface of the carbon fiber layer, starting from the weld. The epoxy resin and curing agent were mixed at a ratio of 3:10 10–30 min before the winding work. After laying the glass fiber layer, the coating operation of the epoxy resin and curing agent mixture was immediately performed to ensure the resin and curing agent fully penetrated the fiber layer, forming a dense and uniform composite structure. During the coating process, tools such as scrapers and rollers shall be used to distribute the resin evenly, and entrapped air bubbles shall be removed in a timely manner to prevent defects like hollowing and delamination from affecting the repair quality. Then, a hot air gun set at 35 °C was used for heating-assisted curing of the layer for no less than 2 h to accelerate the initial cross-linking reaction of the resin and achieve gel surface drying. Finally, sealing tape was wrapped around the repair layer to prevent resin dripping during curing. After the composite had fully cured for at least 24 h, the tape was removed. The excess fiber tape beyond the repair area was then trimmed off with scissors, and the iron mesh was removed.

#### 2.2.3. Bursting Test

**Figure 6:**
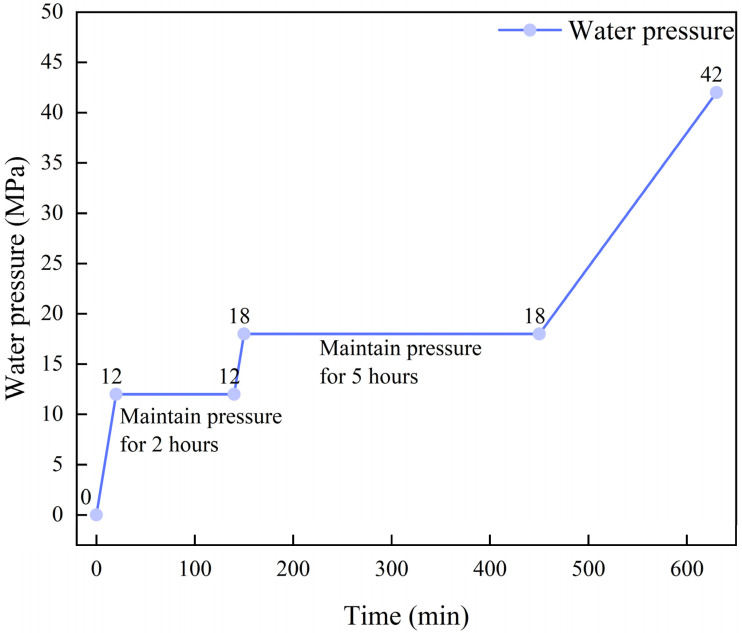
The designed pressure curve in the burst test.

Pressure-bearing capacity is the core indicator for measuring the service quality and structural integrity of pressure vessels. As a key verification method for evaluating the repair effect of composite materials, burst tests can directly reflect the ultimate bearing capacity and failure modes of the repaired pipes [24,25,26]. In accordance with the requirements of standard GB 50251-2015 [23], the pipe must meet the sealing requirements under the design pressure after repair, and the hydrostatic strength test pressure shall not be less than 1.5 times the design pressure. Based on this, this study designed a stepped pressure-boosting blast curve for the repaired pipe fittings, as shown in Figure 6: first, maintain the pressure at the design pressure (12 MPa) for 2 h, then extend the pressure maintenance to 5 h at 1.5 times the design pressure (18 MPa) to fully verify the sealing performance and structural stability of the repair area; if there is no leakage or other abnormalities, continuously increase the pressure until the pipe bursts to obtain its ultimate bearing pressure. Before the pressure is increased to 30 MPa, it is raised at a rate of 0.5 MPa/min. After reaching 30 MPa, the pipe begins to enter the plastic deformation stage, and the pressurization rate is reduced to 0.06–0.08 MPa/min. There is one full-size specimen for each type of defect and one specimen of a defect-free pipe. Strain gauges are attached to the pipe surface near the repair layer, with one strain gauge on the main pipe and one on the branch pipe. The experimental temperature is maintained at 25 °C. During the test, strain gauges were attached to key areas of the pipe body to monitor the strain response under different pressure levels in real time, thereby accurately judging the transition characteristics between elastic/plastic expansion stages of the pipe during the pressure-rising process and providing data support for the quantitative evaluation of the repair effect. The burst test was conducted at Shenyang Guoyi Testing Technology Co., Ltd., Shenyang, China.

## 3. Results and Discussion

### 3.1. Calculation Results and Verification

**Figure 7:**
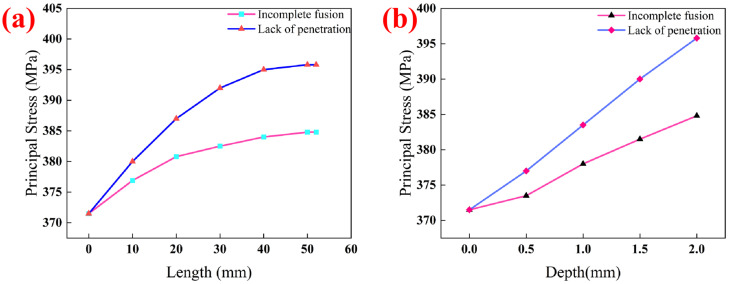
(**a**) The relationship curve of the influence of two types of defect lengths on actual working conditions; (**b**) The relationship curve of the influence of two types of defect depths on actual working conditions.

Figure 7 shows the relationship curves analyzing the influence of the depth and length of incomplete fusion and incomplete penetration defects (taken as single variables) on the bearing performance of pipes under actual service conditions. It can be seen from the figure that the existence of both types of defects will reduce the effective bearing thickness of the fillet weld, thereby aggravating the stress concentration effect in the inner intersection area of the pipe. Notably, the weakening effect of incomplete penetration defects on the bearing strength of the fillet weld is significantly stronger than that of incomplete fusion defects, and the dominant factor affecting the strength of both internal defects is the defect depth. When the defects are symmetrically distributed along the central axis of the main pipe and located in the central area of the pipe’s inner intersection, with the increase in defect length, the principal stress in the fillet weld area first shows a sharp upward trend, and the subsequent growth rate gradually slows down; when the defect length approaches 52 mm, its influence on the bearing performance of the fillet weld tends to stabilize. The essence of this phenomenon is that when the defect length extends beyond the stress concentration area of the fillet weld, the stress level borne by the defect itself decreases significantly, and its impact on the overall bearing performance of the pipe no longer continues to aggravate.

Based on the stress and plastic strain distribution characteristics of the oil and gas pipeline substrate, a calculation study was conducted on the thickness of the composite repair layer required for repairing two typical welding defects (incomplete fusion and incomplete penetration). Considering the influence of the law of defect size on the weld bearing strength, the geometric parameters of both types of defects were uniformly set as follows: depth of 2 mm and length of 1/4 of the pipe circumference (corresponding to a linear dimension of 52 mm). The calculation and analysis results show that the thickness of the composite repair layer required for repairing incomplete penetration defects is 5.6 mm, while that for repairing incomplete fusion defects is 3.2 mm. The determination of the repair layer thickness is based on a comparison of the following three parameters between the repaired pipeline and an undamaged, normally operating pipeline: the magnitude of stress concentration at the defect, the degree of plastic deformation at the defect, and the ultimate pressure-bearing capacity of the pipe component. The specific values are shown in Table 4.

**Figure 8:**
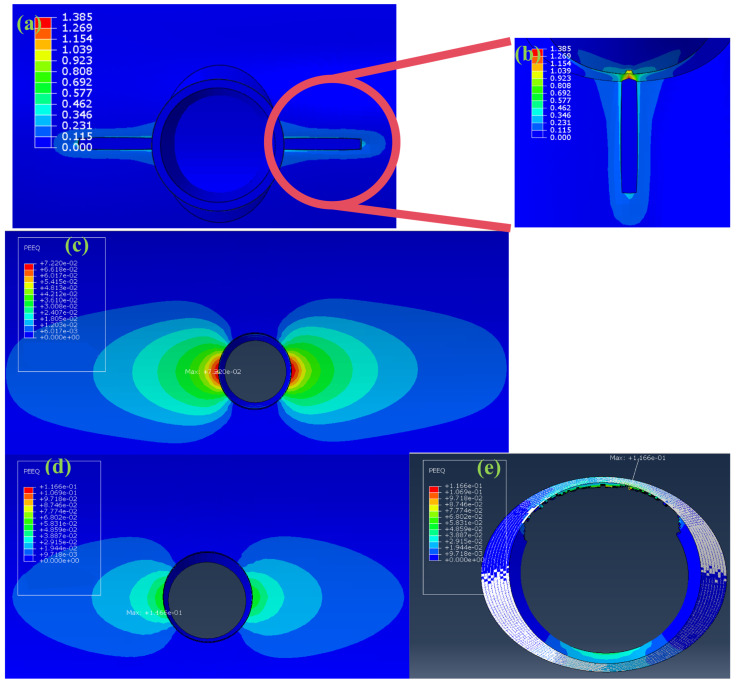
(**a**) Damage morphology of fillet welds in normal pipelines under a load pressure of 34.5 MPa; (**b**) Enlarged view of the failure morphology of fillet welds in normal pipelines under a load pressure of 34.5 MPa; (**c**) Cloud map of strain distribution at the intersection of pipelines with incomplete fusion defects after repair under a load pressure of 35 MPa; (**d**) Cloud map of strain distribution at the intersection of pipelines with incomplete penetration defects after repair under a load pressure of 35 MPa; (**e**) Strain distribution cloud map of fillet welds under a load pressure of 35 MPa after repairing pipelines with incomplete penetration defects.

To verify the rationality of the above repair layer thickness design, firstly, through analysis, the ultimate burst pressure of defect-free pipes under normal working conditions was obtained as 34.5 MPa. Subsequently, 35 MPa, which was greater than this ultimate pressure, was used as the loading condition to verify the anti-destruction performance of the pipes after defect repair, and a comparative analysis was conducted on the deformation characteristics of the weld area of pipes after repairing incomplete fusion and incomplete penetration defects. As shown in Figure 8a,b, for the normal defect-free pipe under a load of 34.5 MPa, its maximum deformation area is concentrated at the fillet weld. The typical plastic instability and rupture behavior induced by pressure overload occur in the connection area between the main pipe and branch pipe near the fillet weld, with the maximum strain value in this area reaching 1.385. For the two types of defect-containing pipes repaired with composite materials, under a pressure load of 35 MPa, the maximum strain value of the pipe after repairing the incomplete fusion defect is 0.0722, and that after repairing the incomplete penetration defect is 0.1166. Moreover, the maximum deformation area of the pipe after repairing the incomplete fusion defect is located at the inner intersection of the pipe, rather than the key bearing area of the fillet weld, which effectively avoids the leakage risk in the weld area.

The above verification results show that the composite reinforcement structure exhibits excellent repair effect: on the one hand, it can effectively achieve the efficient transfer of the pressure load borne by the pipe substrate to the repair layer, reducing the stress level in the key areas of the substrate; on the other hand, it significantly increases the equivalent effective thickness of the defect repair area, ensuring the bearing safety of the pipe.

Considering that the calculations are all based on ideal conditions, there may be improper control in actual operations, and it is necessary to add a safety margin for actual production, the area within 150 mm from the defect on the main pipe wall and within 100 mm from the defect on the branch pipe wall is defined as the core repair area. The actual repair thickness within the core repair area is twice that of the calculated value.

### 3.2. Results of the Blasting Test Verification

**Figure 9:**
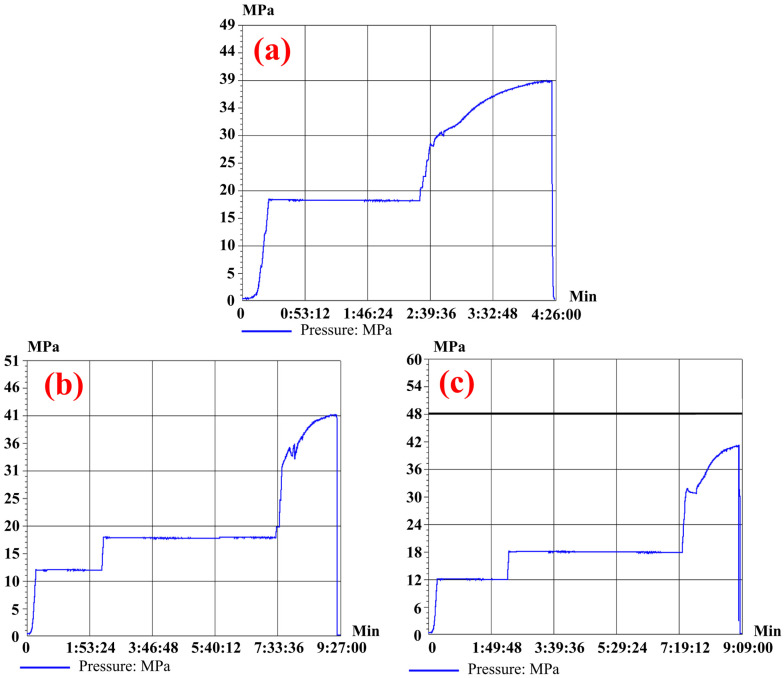
(**a**) Actual pressure curve of normal non-destructive pipeline blasting test; (**b**) Actual pressure curve of burst tests on post-repaired pipelines with incomplete fusion defects; (**c**) Actual pressure curve of pipeline burst test after repair of incomplete penetration defect.

**Figure 10:**
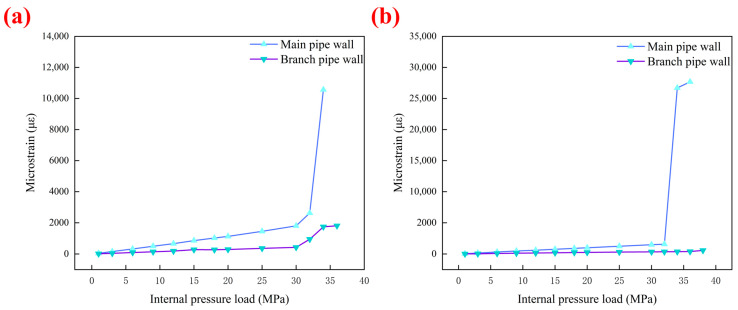
(**a**) Internal pressure-microstrain curve of a pipe with incomplete fusion defect; (**b**) Internal pressure-microstrain curve of a pipe with incomplete penetration defect.

The actual pressure curve of the burst test is shown in Figure 9, and the actual strain curve of the pipe body is shown in Figure 10. It can be seen from Figure 9a that the slope of the pressure curve of the normal pipe is large before approximately 30 MPa, with a fast pressure-rising rate. According to the circumferential internal stress formula for thin-walled pipes in (1) [27], the circumferential internal stress of the main pipe wall when a 30 MPa load is applied is 388.68 MPa, which is similar to the yield strength of L360 steel. Therefore, the pipe is in the stage dominated by elastic expansion at this time, with small pipe body deformation and easy pressure rise. After 30 MPa, the slope decreases and gradually tends to be flat, and the pipe substrate converts to the stage dominated by plastic expansion. Finally, the burst pressure of the normal undamaged pipe is 39.27 MPa.

(1) $$\sigma_{h}\;=\;\frac{P\;\times \;D}{2\;\times \;t}$$

Here, σ_h_ is the hoop stress (MPa), P is the internal pressure load (MPa), D is the pipe diameter (mm), and t is the pipe wall thickness (mm).

Similarly, it can be seen from Figure 9b,c and Figure 10 that for the pipes with two types of internal defects, the pressure-rising rate decreases and the deformation increases suddenly at approximately 32 MPa. This proves that compared with the normal undamaged pipes, the transition interval from elastic deformation to plastic deformation of the pipes repaired by composite reinforcement has increased by approximately 2 MPa. This increase of approximately 2 MPa in the elastic-plastic transition interval is of significant engineering importance for repair quality. Firstly, it signifies a “Delayed Yielding” effect. The high-modulus carbon fiber layer effectively constrains the radial expansion of the steel pipe during the initial loading stage. By sharing the hoop stress, the composite reinforcement keeps the steel substrate within its elastic range for a wider pressure span. Secondly, extending the elastic range enhances the operational safety margin. It implies that the repaired pipeline can withstand higher pressure fluctuations (surges) without undergoing permanent plastic deformation. While the ultimate burst pressure indicates the failure limit, the elevated transition point demonstrates that the repair structure actively contributes to stiffness and structural integrity well before the critical failure state is reached.

**Figure 11:**
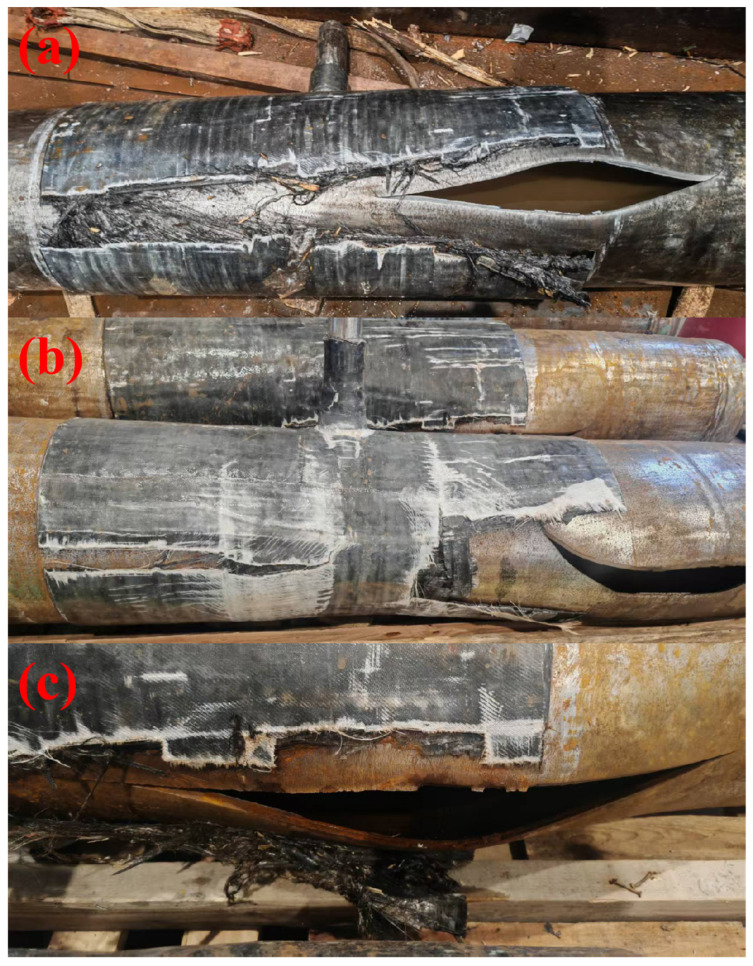
(**a**) Failure morphology of pipe bodies with incomplete penetration defects after burst tests; (**b**) Failure morphology of pipe bodies with incomplete fusion defects after burst tests; (**c**) Damage morphology of composite repaired structures.

Figure 11 shows the failure morphology of the pipe after the burst test. When the normal defect-free pipe reaches its ultimate bearing capacity, it first leaks from the fillet weld. The failure location of the repaired pipe is on the pipe body in the non-core repair area where the repair layer is relatively thin. Through “stress redistribution-cooperative bearing”, the reinforcement layer strengthens the original “fillet weld defect weak area” into a “high-strength composite area”, naturally transferring the weak point of the structure to the pipe body that is relatively close to the stress concentration area of the fillet weld and has relatively low strength due to the attenuation of the repair layer thickness. Among them, the burst pressure of the pipe with incomplete penetration defects after repair reaches 41.03 MPa, and that of the pipe with incomplete fusion defects after repair reaches 40.84 MPa. This achieves the goal of successfully repairing the welding defects of the branch pipe fillet weld of oil and gas pipelines by means of composite reinforcement.

The observed shift in failure location is governed by the stress shielding mechanism resulting from the stiffness mismatch between the high-modulus carbon fiber layer and the steel substrate. During pressurization, the composite layer acts as a rigid constraint, effectively suppressing the radial expansion of the defect-prone weld area. This interaction redistributes the hoop stress from the internal weld root (the stress concentration point) to the composite reinforcement layer. Consequently, the “weakest link” of the system shifts from the internal welding defect to the adjacent pipe body in the non-core repair area, where the reinforcement thickness attenuates. The final failure occurs in the pipe body because the localized stress exceeds the ultimate tensile strength of the L360 steel, while the reinforced weld area remains within a safe stress range due to the effective load transfer.

Limitations and Future Work Despite the demonstrated effectiveness of the GF-CF-GF composite repair system, this study has certain limitations that should be addressed in future research. First, the experimental validation in this study focused primarily on the static ultimate bursting pressure under ambient temperature conditions. The long-term fatigue performance of the repaired branch pipes under cyclic pressure fluctuations, as well as the durability of the bonding interface under complex environmental factors (such as hygrothermal aging and chemical corrosion), remains to be quantitatively assessed. Furthermore, due to the high cost and destructive nature of full-scale burst testing, only one specimen per defect type was tested in this study. While this limited sample size prevents a comprehensive statistical analysis of experimental scatter and repeatability, the distinct shift in failure mode—from the weld defect to the pipe body—provided robust deterministic evidence of the repair’s effectiveness. Second, the internal defects modeled and tested in this study were idealized as regular geometric shapes (uniform depth and length) for the sake of variable control. In actual engineering scenarios, welding defects often possess irregular morphologies, which may induce more complex stress concentration behaviors. Future work will focus on investigating the fatigue life of such repaired structures and developing more refined damage models that account for irregular defect topologies, as well as the durability of the bonding interface under complex environmental factors (such as creep, temperature cycling, and chemical corrosion).

## 4. Conclusions

This study addresses the critical challenge of repairing internal defects (incomplete fusion and penetration) in small-diameter branch pipe fillet welds by proposing and validating a GF-CF-GF sandwich composite reinforcement system. By integrating finite element analysis with full-scale burst testing, the following conclusions are drawn:

(1) Effective Structural Design Methodology: A systematic design method for identifying optimal repair parameters was established. The numerical analysis revealed that defect depth is the dominant factor affecting the bearing capacity of fillet welds, more so than defect length. Based on this insight, the critical repair thicknesses were determined (5.6 mm for incomplete penetration and 3.2 mm for incomplete fusion) to ensure the stress level in the defect area is reduced to a safe range.
(2) Mechanism of Performance Enhancement: The burst tests confirmed that the composite repair successfully shifted the failure mode from the brittle fracture of the weld root to the ductile failure of the pipe body. Crucially, beyond merely recovering the ultimate burst pressure (reaching >40 MPa), the repair extended the elastic-plastic transition interval by approximately 2 MPa. This synthesis of results indicates that the high-modulus carbon fiber layer provides significant radial constraint and delays the yielding of the steel substrate, thereby enhancing the safety margin against pressure surges.
(3) Feasibility for In-Service Application: The proposed GF-CF-GF sandwich structure effectively isolates the carbon fiber from the steel, mitigating galvanic corrosion risks while accommodating the complex saddle-shaped geometry. The standardized winding process and pressure testing curve developed in this study provide a verified, low-cost, and non-hot-work solution for the maintenance of oil and gas station pipelines.

## Figures and Tables

**Table 1:** L245 and L360 pipeline steel material properties.

Material	Density (g/cm^3^)	Yield Strength (MPa)	Elastic Modulus (MPa)	Poisson’s Ratio	Fracture Train	Triaxial Tress	Strain Atio	Destruction Isplacement
L245	7.85	245	200,000	0.3	0.15	0.33	1.1	7.5
L360		360						

**Table 2:** GF-CF-GF sandwich design, 35 °C cured composite material performance parameters.

Material	Composite Material
Density (g/cm^3^)	1.77
Modulus (GPa)	20.7
Poisson’s ratio	0.2
Longitudinal tensile strength (MPa)	487
Longitudinal compressive strength (MPa)	230
Transverse tensile strength (MPa)	400
Lateral compressive strength (MPa)	220
Longitudinal shear strength (MPa)	110
Lateral shear strength (MPa)	104
Winding angle	45°/0°/−45°/90°
Cure temperature	35 °C

**Table 3:** Finite Element Setup Table.

Types of Finite Element Setting Parameters		Input Parameters
Maximum time increment step		3 × 10^7^
Quality Scaling: Factor		100
Load Type		Pressure
Load (MPa)		18
Unit library		Explicit
Geometry period		Linear
Abaqus/Explicit Precision		Analysis + packager
Hashin Damage	Longitudinal tensile fracture energy	16
	Longitudinal compressive fracture energy	10
	Transverse tensile fracture energy	0.22
	Transverse compressive fracture energy	1.1
Johnson-Cook Damage	Fracture strain	0.15
	Triaxial stress	0.33
	Strain Ratio	1.1
Element Deletion Criterion (Dmax)		1.0
Softening Law		Linear

**Table 4:** Comparison of calculation results between repaired pipelines and undamaged in-service pipelines (the load is 1.5 times the operating pressure, 18 MPa).

Data Type	Pipe Condition		
	Normal Service Pipeline	Pipes with Incomplete Fusion Defects (After Repair)	Pipes with Incomplete Penetration Defects (After Repair)
Maximum Mises stress at the fillet weld (MPa)	371.5	364.0	364.7
Maximum plastic deformation (PEEQ) at the fillet weld	3.505 × 10^−3^	3.273 × 10^−3^	1.638 × 10^−3^
Maximum Load Capacity (MPa)	34.5	36.4	35.9

## Data Availability

The original contributions presented in this study are included in the article. Further inquiries can be directed to the corresponding authors.

## References

[1] Luo Y., Jiao X., Fang Z., Zhang S., Wu X., Wang D., Chu Q. (2021). Remote deepwater subsea pipeline maintenance system. Ind. Robot Int. J. Robot. Res. Appl..

[2] Wang G., Ren G., Deng B., Zhang L., Jia P. (2023). Verification Analysis of X70 Type B Sleeve for In-service Pipeline Repair. J. Fail. Anal. Prev..

[3] Baek J.H., Jang Y.C., Kim I.J., Yoo J.S., Kim C.M., Kim Y.P. (2021). Influence of welding processes and weld configurationon fatigue failure of natural gas branch pipe. Int. J. Press. Vessel. Pip..

[4] Liu H., Zhou S., Gu W., Zhuang W., Gao M., Chan C.C., Zhang X. (2025). Coordinated planning model for multi-regional ammonia industries leveraging hydrogen supply chain and power grid integration: A case study of Shandong. Appl. Energy.

[5] Qiao Y., Bian X., Ke H., Zhao Z., Zhang X., Zhang X., Zhao Y., Bai C., Zhang L., Zheng T. (2025). Phenolic-based porous composite with embedded short carbon fiber/hollow spheres for mechanical properties and thermostability. Compos. Commun..

[6] Ariff I.M., Wong K.J., Gan K.W., Israr H.A., Tamin M.N. (2025). Progressive damage analyses of patch repaired composites using acoustic emission and digital image correlation. Int. J. Adhes. Adhes..

[7] Zhang X., Ke H., Qiao Y., Zhang X., Zhang L., Bai C., Lin L., Shao K., Nan H., Wang X. (2025). High-performance chopped carbon fibers reinforced epoxy adhesive for underwater structural repair and strengthening applications. Structures.

[8] Zhou X., Wu M., Li Y., Qiao Y., Wang P., Xie B., Wang B., Wang Z., Zhou J., Chen S. (2025). Repair process of fillet weld defects in oil and gas branch pipes with composite materials: Finite-element modelling and experimental verification. Mater. Technol..

[9] Xu G., Wang K., Zhen Y., Shen Y., Han F., Wang H., Yue X., Hou J., Jiang H., Ding W. (2024). A FeCrB-based composite containing multi-scale corrosion-resistant phases: Application in high-temperature liquid aluminum corrosion. J. Mater. Res. Technol..

[10] Di Z., Wang Y., Chang C., Song H., Lu X., Cheng F. (2025). Synergistic gas–slag scheme to mitigate CO_2_ emissions from the steel industry. Nat. Sustain..

[11] Shen G., Liu L., Zheng G., Xia Y. (2025). Implementation of the peridynamics-based finite element method of thin shells for brittle fractures in Abaqus. Eng. Fract. Mech..

[12] Zhou Y., Xu K., Geng H., Jiang J., Guo X., Zhang C. (2025). Novel heat-resistant and tough cyanate ester resin matrix for carbon fiber composite via wet winding process molding at a reduced temperature. Polym. Compos..

[13] Wang A., Liu X., Yue Q., Xian G. (2023). Tensile properties hybrid effect of unidirectional flax/carbon fiber hybrid reinforced polymer composites. J. Mater. Res. Technol..

[14] Tan Z., Li H., Yu J., Yan S., Ren K., Wang Z. (2025). Design Optimization and Experiments of Composite Structure Based Pressure Hull for Full-Ocean-Depth Underwater Vehicles. J. Mar. Sci. Eng..

[15] Zhang Y., Van Paepegem W., De Corte W. (2024). An Enhanced Progressive Damage Model for Laminated Fiber-Reinforced Composites Using the 3D Hashin Failure Criterion: A Multi-Level Analysis and Validation. Materials.

[16] Alexandrov S., Strashnov S., Li Y. (2023). An Upper Bound Solution for Axisymmetric Extrusion and Drawing Considering a Generalized Yield Criterion. Metals.

[17] Ma Y., Ma W., Li M., Liu Q., Luo S., Wang Y., Lei C. (2025). Research on the structural strength of medium and low speed maglev vehicle levitation frame based on full-scale test bench. Eng. Fail. Anal..

[18] Bhardwaj U., Teixeira A.P. (2021). Guedes Soares, Burst strength assessment of X100 to X120 ultra-high strength corroded pipes. Ocean Eng..

[19] Lee S.H., Abolmaali A., Shin K.J., Lee H.D. (2020). ABAQUS modeling for post-tensioned reinforced concrete beams. J. Build. Eng..

[20] Wei Z., Dong P., Pei X. (2023). The structural strain method for fatigue evaluation of welded components: Closed-form solutions. Int. J. Fatigue.

[21] He S., Wang K., Zhao Y., Lu Y., Jiang R., Li J., Xiang Y. (2025). Comparative analysis of corrosion resistance of pipeline steels exposed to sulfate-reducing bacteria: Insights on L360, L245NS and antibacterial steels. Int. J. Electrochem. Sci..

[22] Yang M., Tang J., Kainuma S. (2025). Inhibition and facilitation mechanisms of galvanic corrosion between carbon fiber and steel in atmospheric environments. Compos. Part B Eng..

[23] Knysh V.V., Mordyuk B.N., Solovei S.O., Savitsky V.V., Mikhodui O.L., Lesyk D.A., Motrunich S.I. (2024). HFMI-induced fatigue strength improvement of S355 steel transverse non-load-carrying attachments with lack of fusion in the weld root. Int. J. Fatigue.

[24] Guillon D., Espinassou D., Pichon P., Carrillo J.J.R., Landry C., Clainchard D., Juras L., Brault R. (2023). Manufacturing, burst test and modeling of high pressure thermoplastic composite overwrap pressure vessel. Compos. Struct..

[25] Yang C., Jiang P., Li W., Zuo K., Duan B. (2025). Qualitative and quantitative damage assessment of composite pressure vessels on the basis of acoustic emission parameters. Polym. Compos..

[26] Bouhala L., Polesel J., Karatrantos A., Perbal S., Senf B., Hiekel A., Reinhardt H., Rauscher A., Mäder T. (2025). Review of State-of-the-art of structural health monitoring in hydrogen composite pressure vessels. Compos. Part C Open Access.

[27] Chen H.J., Zheng Z.J., Wen H.M. (2025). A numerical and analytical study on the burst pressure of a defective pipeline. Int. J. Press. Vessel. Pip..

